# Improved antibiotic stewardship resulting from a multifaceted strategy implemented after an outbreak of multiresistant *Acinetobacter baumannii *in a university ICU

**DOI:** 10.1186/cc10653

**Published:** 2012-03-20

**Authors:** M Beach, M Cohen, V Grover, J Ho, N Soni, B Azadian, S Singh

**Affiliations:** 1 Chelsea & Westminster Hospital, London, UK

## Introduction

A 12-bed ICU experienced an outbreak of multiresistant *Acinetobacter baumannii *(MRA) from October 2009 to May 2010. A multifaceted strategy involving segregation, enhanced infection control procedures, and microbiological surveillance was implemented. We evaluated its impact on antibiotic stewardship.

## Methods

A retrospective review of patient notes and results using AcuBase^® ^was conducted: 90 consecutive patients before the outbreak (January to June 2008) and 91 thereafter (October 2010 to May 2011). Data included patient profiles, admission criteria, ICU survival, antimicrobials used, antibiotic days, number of patients on antibiotics, prescribing cost and the demographic of microbes isolated.

## Results

**Figure 1:**
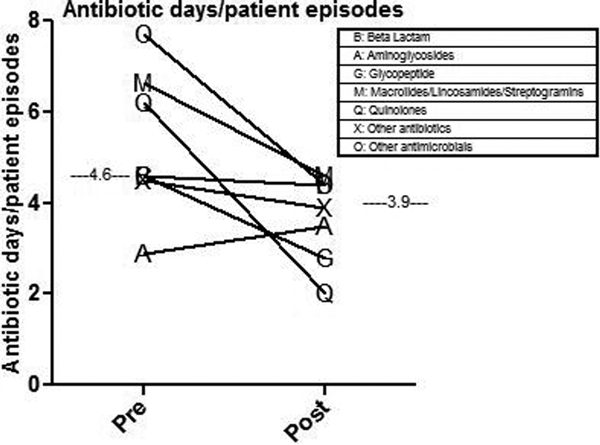


Following the outbreak, enhanced infection control measures were implemented alongside the Matching Michigan protocols. Daily operational critical care and elective planning meetings and a staff education programme were undertaken. ICU mortality (31 (14%) vs. 43 (16%)) was unchanged. Microbiological isolates were overall similar, with a reduction in coagulase-negative *Staphylococcus *and *Klebsiella *and an increase in *Enterobacter*. The use of cefuroxime (3.2 vs. 2.3 antibiotic days/patient) and quinolones (6 vs. 2) decreased. There was a reduction in average antibiotic days per patient episode (5.1 vs. 4.2) (*P *= 0.0291) and the prescribing cost savings were £13,558 (47%). See Figure 1.

## Conclusion

The need for a complex strategy to manage and eradicate an MRA outbreak in the ICU led to a clinically significant decrease in antibiotic use and prescribing cost saving following eradication of MRA. Improved antibiotic stewardship is achievable through better infection control strategies.
